# Timing for Ponseti clubfoot management: does the age matter? 90 children (131 feet) with a mean follow-up of 5 years

**DOI:** 10.1080/17453674.2018.1526534

**Published:** 2018-10-18

**Authors:** Yu-Bin Liu, Song-Jian Li, Li Zhao, Bo Yu, Da-Hang Zhao

**Affiliations:** 1 Department of Orthopaedics, Zhujiang Hospital of Southern Medical University, Guangzhou;;; 2 Ying-Hua Medical Group of Bone and Joint Healthcare in Children, Shanghai;;; 3 Department of Pediatric Orthopaedics, Xin-Hua Hospital affiliated to Shanghai Jiao Tong University School of Medicine, Shanghai, China

## Abstract

Background and purpose — There are still controversies as to the age for beginning treatment with the Ponseti method. We evaluated the clinical outcome with different age at onset of Ponseti management for clubfoot.

Patients and methods — 90 included children were divided into 3 groups in terms of age at start of treatment. The difference in treatment-related and prognosis-related variables including presentation age, initial Pirani and Dimeglio score, casts required, relapse rates, final Dimeglio score, and international clubfoot study group score (ICFSG) was analyzed.

Results — Age between 28 days and 3 months at start of treatment method was associated with fewer casts required, lower relapse rate, and lower final ICFSG score (p < 0.05). Early treatment before 28 days of age required more casts and had a higher relapse rate (p < 0.05). The highest ICFSG scores were found in the ages between 3 and 6 months (p < 0.05). After propensity score matching, age between 28 days and 3 months was demonstrated to have a lower finial ICFSG score. Linear regression models showed that presentation age was positively correlated with final ICFSG score, and was identified as the only independent prognostic risk factor.

Interpretation — There was lower rate of relapse and better clinical outcome when treatment was initiated at age between 28 days and 3 months. With the Ponseti method, clubfeet may not need urgent treatment.

Idiopathic clubfoot appears with an incidence of 5/10^4^ in China (Yi et al. 2013). Nowadays, the Ponseti method has been widely accepted as the treatment of choice, and its safety and efficacy has been extensively demonstrated around the world (Zhao et al. 2014a, Liu et al. 2016). Generally, treatment with the Ponseti method is started within the first few weeks of life (Ponseti 1996, Dobbs and Gurnett 2009, Zhao et al. 2014b, Liu et al. 2016). In the study by Alves et al. (2009), the patients were divided into 2 groups (younger or older than 6 months) and no difference was found in the number of casts, tenotomies, success in terms of rate of initial correction, rate of recurrence, or rate of tibialis anterior transference. Iltar et al. (2010) reported that casting treatment beginning later than age 1 month or with an affected foot ≥8 cm in length had better treatment outcome. Zionts et al. (2016) reported that the age at the onset of treatment did not appreciably influence the cast phase of treatment or the initiation of post-corrective bracing, with the exception of cast slippage. Awang et al. (2014) have demonstrated that the total number of castings required to treat clubfoot was determined by the severity of clubfoot but not by the weight and age of patients. Other authors have reported that children presenting late with clubfoot can also be successfully treated by the Ponseti method (Bor et al. 2006, Lourenco and Morcuende 2007, Haj Zargar Bashi et al. 2016). It is still unclear if the treatment outcome is related to the age when the treatment was initiated. We assessed whether age at start of treatment influences the number of casts, tenotomies, the correction rates, recurrence rates, ankle dorsiflexion after treatment, final Demeglio and international clubfoot study group score (ICFSG) with a mean follow-up of 5 years.

## Patients and methods

Children who had been diagnosed with idiopathic clubfeet, and who were treated with the Ponseti method, were retrospectively reviewed in the study. The inclusion criteria entailed the following: first presented in our hospital at age less than 6 months, finished the whole protocol of the Ponseti method, and at least 4 years of post-treatment follow-up. Children were excluded from this study for the following reasons: postural, syndromic, and neurological clubfeet; previous treatment before referral; lost to follow-up at our institution before reaching 4 years of age; data lost or incomplete; declined to participate.

**Figure 1.**
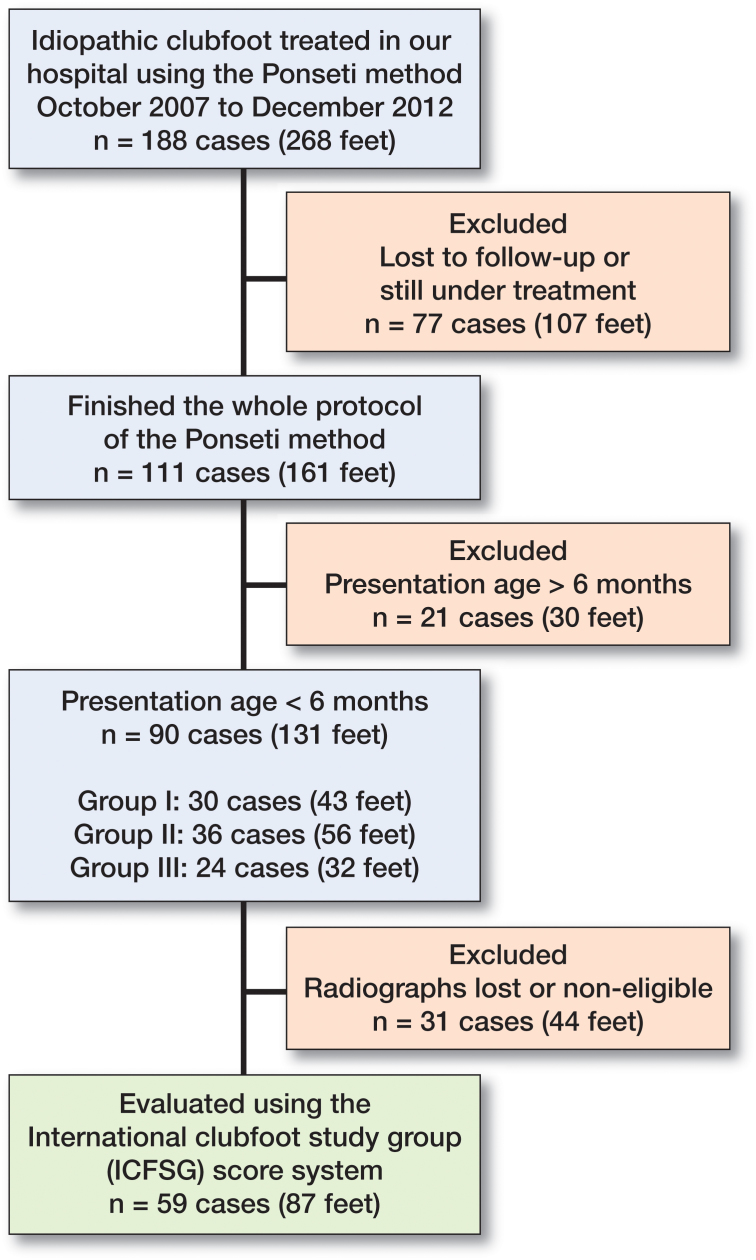
Flowchart of patients with idiopathic clubfoot.

We reviewed the records of 188 patients (268 idiopathic clubfeet) treated consecutively between October 2007 and December 2012. 98 children were excluded from this study for the reasons outlined in Figure 1. 90 children (131 clubfeet) were divided into 3 groups according to the age at initiation of treatment, Group I (the initial treatment age younger than 28 days), Group II (age more than 28 days but less than 3 months) and Group III (age more than 3 months but less than 6 months). For each case included in the study, the gestational and presentation age, sex, side of clubfoot, Pirani and Dimeglio scores were recorded before initial treatment.

**Figure 2.**
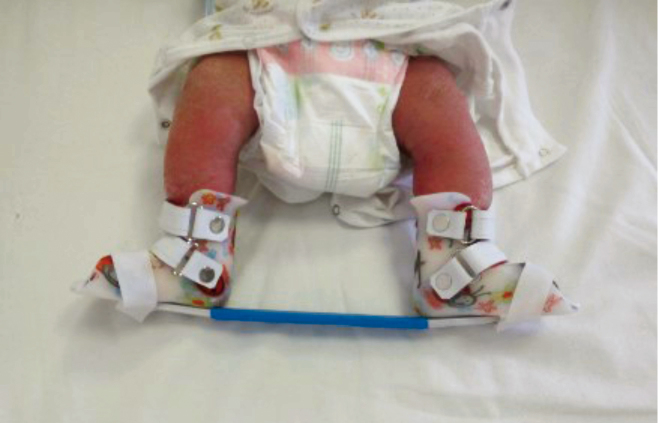
Foot-abduction brace was applied in our clinics. The brace consists of a bar with the shoes attached at 60–70° of abduction on the affected side and 30–40° on the normal side.

We strictly followed the protocol outlined in the Ponseti method. All the clubfoot cases were treated by a single orthopedist (LZ). A long leg cast was applied after the first evaluation in the outpatient clinic. All children were treated without anesthesia or sedative. The special technician in our clinics was responsible for the removal of the cast, and parents were not recommended to remove the cast themselves. Evaluation using Pirani score and Demeglio score was performed each time after removal of the cast. After the last cast, the procedure of percutaneous Achilles tenotomy (PAT) is indicated if ankle dorsiflexion is less than 15° and the foot abducted to 60–70° without pronation. Post PAT long-leg cast was applied for 3 weeks. When the full correction had been achieved, a foot abduction orthosis (FAO, Figure 2) was prescribed to maintain the foot in the corrected position. The brace protocol was in full-time use for the first 3 months, then 16 to 18 hours until the children were 2 years old, then 14 to 16 hours until 4 years old. Relapses were treated with repeated manipulation and casting with or without PAT, followed by the use of the foot-abduction brace. Tibialis anterior tendon transfer (TATT) was performed in children with dynamic supination of the forefoot during the swing phase of gait. Wearing of the brace was required for an additional year after the removal of the last cast in relapsed cases identified at older than 4 years of age. We defined relapse as recurrence of any component of the deformities including adductus, varus, cavus, and equinus requiring further intervention, including either repeated manipulation and casting or surgical intervention. Noncompliance was defined as not wearing the brace for at least 75% of the number of hours prescribed (Liu et al. 2016), as reported by the parents. The mean follow-up was 5 years (4–8).

## Statistics

Comparisons of 3 groups in terms of Pirani and Dimeglio scores, number of casts before PAT, ankle dorsiflexion degrees, mean follow-up, and ICFSG scores were performed using ANOVA. The chi-square test for comparison of multiple rate among 3 groups was applied for comparing variables such as the rate of PAT, initial correction rate, compliance rate, and relapse rate. Univariable and stepwise multivariable linear regressions were employed to estimate the correlations of each clinical characteristics with final ICFSG scores.

The propensity scores were estimated using a logistic regression model that included the following eight covariates: gender, bilateral or unilateral, initial Pirani score, initial Dimeglio score, number of casts before PAT, PAT or not, brace compliance, and relapse or not. The matching approach was 1:1 nearest neighbor. Absolute standardized differences for all baseline covariates of <10% were accepted as adequate balance. All statistical analyses were performed with SPSS® (IBM SPSS Statistics, version 24; IBM, Armonk, NY, USA) and twang R library (available at: cran.r-project.org/web/packages/twang/index.html). A 2-tailed test with p < 0.05 was considered significantly different.

## Ethics, funding, and potential conflicts of interest

Informed consent was obtained from all the parents. This study was approved by the institutional ethics committee (approval number XHEC-D-2017-061) and was performed in accordance with the Declaration of Helsinki. This work was supported by National Natural Science Foundation of China (No. 81802215). No competing interests were declared.

## Results

Of 188 cases (268 feet), 61 cases (86 feet) were lost to follow-up with mean brace-wearing time of 11 months (ranges, 0–40 months), and 16 cases (21 feet) were still under treatment due to relapse. Noncompliance was associated with 3.3 times greater odds of relapse (16 of 33) in comparison with compliance (14 of 94). The included 90 cases (131 feet) that had finished the whole recommended protocol of the Ponseti method and were followed up until 4 years of age with complete information collected in our database. There were no preterm infants, and the mean gestational age was 39.4 weeks (range, 37–41 weeks) at birth.

**Figure 3.**
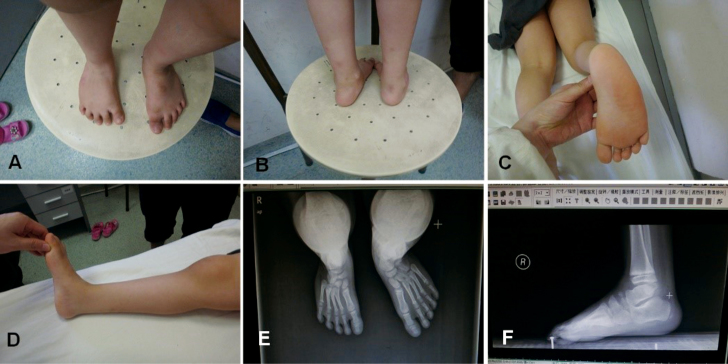
Relapse was identified at 4 years of age for poor brace compliance. Right clubfoot: Dimeglio score =8. (A) Forefoot adductus, (B) No obvious varus deformity, (C) Lateral edge curve, (D) Equinus deformity, (E) Standing anteroposterior views: reduced talocalcaneal angle, (F) Standing lateral views: reduced talocalcaneal angle and flat-top talus.

Of the 90 cases (131 feet), the mean age at the onset of treatment was 48 days (2 days to 6 months) with a mean Pirani score of 4.5 points (2.5–6) and a mean Dimeglio score of 13 points (6–17). 78 children (115 feet) had the PAT procedure, and the initial correction rate was 87%. Cast slippage was observed in 3 cases (4 feet) for Group I with severe equinus deformity. No skin necrosis or ulcer, rocker bottom deformity, or neurovascular compromise post-tenotomy were observed. 14 children reported the brace was used <75% of the prescribed time. Relapses occurred in 13 children (21 feet) with a mean relapse age of 34 months (11 months to 5 years). The most common relapse was presented with adductus and equinus (5 cases, 8 feet [Figure 3]), followed by adductus (4 cases, 8 feet). Relapses were treated with a second series of manipulation and casting, followed by the use of a foot-abduction brace. 3 children (4 feet) required a second tendo Achilles tenotomy. 1 child (1 foot) required a TATT procedure.

**Figure 4.**
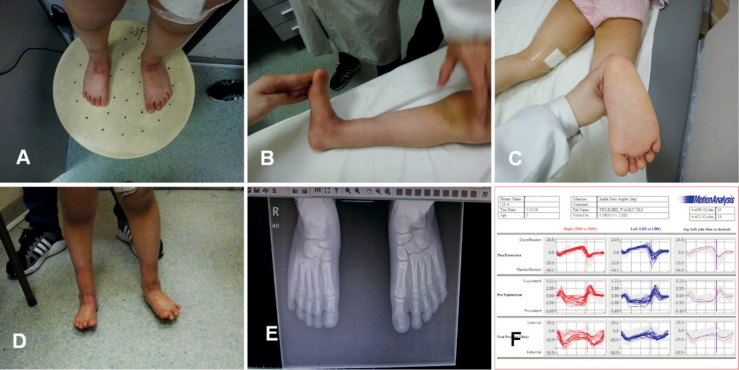
The same patient after repeated Ponseti treatment: ICFSG =4. (A–C) Morphology evaluation =1, (D) Muscle function =0, (E) Radiologic evaluation =3, (F) Gait analysis: dynamic function =0.

59 children (87 feet) were evaluated using the scoring system of the International Clubfoot Study Group score (ICFSG). 31 children (44 feet) were excluded from this evaluation because of non-weight-bearing radiographs. The outcome of clinical evaluation in terms of foot morphology, function, and radiology was evaluated as good or excellent for all 3 groups according to the ICFSG rating system (Figure 4).

The influence of age at the beginning of treatment for clubfoot with Ponseti management is presented in Table 1. There was no statistically significant difference in the severity of clubfoot deformity at initial presentation, as assessed by both the Pirani and Dimeglio scoring systems. A statistically significant difference was found in the number of casts (Group I and Group III) required before the PAT procedure in comparison with Group II. There was no statistically significant difference in the PAT rates, initial correction rates, compliance rates, ankle dorsiflexion degrees after treatment, mean follow-up, and finial Dimeglio score among the three groups. The relapse rate was lower in Group II in comparison with Group I, and a statistically significant difference was found between them (p < 0.05). After completion of the whole protocol of the Ponseti method, the mean scores of Group III were the highest in comparison with other groups (Group I and Group II) with a statistically significant difference between them.

**Table 1.** Demographic data with reference to age when clubfoot management was initiated

	Group I	Group II	Group III
No. of cases (feet)	30 (43)	36 (56)	24 (32)
Age at presentation range	13 days	47 days	4.5 months
	(2–27)	(29–90)	(3–6)
Sex			
Male	25	29	18
Female	5	7	6
Side			
Bilateral	13	20	8
Unilateral	17	16	16
Initial Pirani score^c^	4.8 (0.9)	4.3 (1.1)	4.3 (0.7)
Initial Dimeglio score^c^	14 (3.5)	13 (3.1)	13 (2.7)
No. of casts before PAT^c^	4.5 (1.6)^a^	3.7 (1.2)	4.2 (1.4)^a^
PAT rate^d^			
Yes	28 (40)	29 (46)	22 (28)
No	2 (3)	7 (10)	2 (4)
Initial correction rate^d^			
Yes	22 (33)	33 (51)	20 (26)
No	8 (10)	3 (5)	4 (6)
Brace compliance^d^			
Yes	25 (35)	30 (46)	1 8 (26)
No	5 (8)	6 (10)	6 (6)
Relapse rate^d^			
Yes	7 (14)	4 (6)^b^	2 (2)
No	23 (29)	32 (50)	22 (30)
Dorsal flexion (°)^c^	13.7 (9.2)	15.2 (8.3)	14.8 (7.9)
Mean follow-up (years)^c^	4.9 (1.2)	4.7 (0.7)	4.9 (0.7)
Final Dimeglio score^c^	4.3 (1.3)	4.1 (0.8)	4.1 (0.6)
ICFSG score^d^	24 (34)	21 (35)	14 (18)
Total score^c^	4.6 (1.4)	3.9 (2.2)	6.3 (1.1) ^a,b^

Group I (≤ 28 days), Group II (> 28 days to ≤3 months), Group III (> 3 months to ≤6 months);

a p < 0.05 compared with Group II.

b p < 0.05 compared with Group I.

c values are mean (SD)

d values are cases (feet)

The correlations of clinical characteristics with final ICFSG score were analyzed using univariable and stepwise multivariable linear regression models (Table 2). In the cases with bilateral clubfeet who had different initial Pirani score, Dimegliso score, or required number of casts for correction, the greater score or bigger number was used accordingly. As for univariable linear regression analysis, the results revealed that final ICFSG score was positively correlated with presentation age (p = 0.002), but negatively associated with initial Pirani score and initial Dimeglio score (p = 0.04, p = 0.04, respectively). After the stepwise multivariable linear regression analysis with adjustment for covariates, presentation age was found to be positively associated with final ICFSG score with statistical significance (p < 0.001).

**Table 2.** Linear regression of ICFSG score with clinical characteristics (n = 59)

Variables	Univariable linear regression β (95% CI)	Multivariable linear regression p-value	β (95% CI)	p-value
Presentation age (days)	0.017 (0.007 to 0.027)	0.002	0.019 (0.009–0.029)	< 0.001
Sex	0.18 (–1.3 to 1.7)	0.8	–	–
Side	–0.14 (–1.2 to 0.9)	0.8	–	–
Initial Pirani score	–0.51 (–1.0 to –0.02)	0.04	–0.11	0.4
Initial Dimeglio score	–0.16 (–0.3 to –0.01)	0.04	–0.22	0.08
No. of casts	0.18 (–0.2 to 0.6)	0.3	–	–
PAT	–1.23 (–2.8 to 0.3)	0.1	–	–
Compliance	0.47 (–0.9 to 1.8)	0.5	–	–
Relapse	–0.04 (–1.5 to 1.5)	1.0	–	–

ICFSG: International Clubfoot Study Group score.

For further analysis of the cause–effect relations between presentation age and final ICFSG score, the propensity score matching method was applied to adjust for potential confounding factors. After propensity score matching, the covariates such as sex, bilateral or unilateral, initial Pirani score, initial Dimeglio score, number of casts before PAT, PAT or not, brace compliance, and relapse or not, were well balanced among groups. The results showed that the mean ICFSG scores of Group III were the highest in comparison with the other groups (Group I and Group II) with a statistically significant difference, and Group II was found to have the lowest final ICFSG score after completing Ponseti method treatment (Table 3).

**Table 3.** Demographic data after propensity score matching

	Group I	Group II	Group III
No. of cases (feet)	15 (25)	15 (24)	10 (16)
Age at presentation	13 days	45 days	4.6 months
range	(3–27)	(31–87)	(3–6)
Sex			
Male	12	13	8
Female	3	2	2
Side			
Bilateral	10	9	6
Unilateral	5	6	4
Initial Pirani score ^c^	4.6 (0.9)	4.5 (1.2)	4.1 (0.7)
Initial Dimeglio score ^c^	14 (3.9)	13 (3.9)	14 (1.2)
No. of casts before PAT ^c^	4.3 (1.8)	3.5 (0.9)	4.4 (1.0)
PAT rate ^d^			
Yes	13 (22)	13 (22)	10 (16)
No	2 (3)	2 (2)	0 (0)
Brace compliance ^d^			
Yes	13 (21)	13 (21)	6 (12)
No	2 (4)	2 (3)	4 (4)
Relapse rate ^d^			
Yes	5 (10)	2 (3)	2 (2)
No	10 (15)	13 (21)	8 (14)
Propensity score ^c^	0.5 (0.2)	0.5 (0.2)	0.6 (0.2)
ICFSG score ^c^	4.9 (1.6)	3.9 (2.2)	6.8 (1.1) ^a,b^

For footnotes see Table 1.

## Discussion

The proposal that congenital clubfoot should be treated soon after birth has been widely accepted (Dobbs et al. 2004, Morcuende et al. 2004, Dobbs and Gurnett 2009, Zhao et al. 2014a, Liu et al. 2016). Ponseti (1996) also suggested that initial treatment should begin in the first few weeks of life to take advantage of the more favorable viscoelastic properties of the connective tissues in the newborn. The upper limit age for Ponseti method management is unclear. Several authors have reported that neglected clubfoot cases or patients presenting at an older age could also be successfully managed by the Ponseti method (Lourenco and Morcuende 2007, Spiegel et al. 2009, Khan and Kumar 2010, Ayana et al. 2014). In a recently published study in which 11 neglected patients with a mean age of 11 years (6–19) were treated with a modified Ponseti method, 17 out of 18 feet achieved a good result with no need for further surgery (Haj Zargar Bashi et al. 2016). It is still unknown whether the age at initiation of treatment influences the clinical outcome and the rate of relapse.

We found that start of treatment age between 28 days and 3 months was identified with fewer casts required before PAT procedure, lower relapse rate, and better final ICFSG score. Stepwise multivariable linear regression models identified presentation age as the only independent prognostic risk factor for final clinical outcome. Earlier treatment with presentation age less than 28 days required more casts before the PAT procedure and had a higher relapse rate during the process of Ponseti method management. This finding is in contrast to previous recommendations that treatment should be started soon (7 to 10 days) after birth (Ponseti 1996, Dobbs et al. 2004, Liu et al. 2016). Our finding supports the notion that patients with presentation age after the first month achieved a better clinical outcome than those with earlier presentation (Iltar et al. 2010). The variance was attributed to the long age span in the older infant group (> 30 days, but <1 year of age). The less satisfactory results in newborns may be attributed to small feet and partial Achilles tenotomy. Karami et al. (2015) demonstrated that complete Achilles tenotomy for newborns was identified in only 7/16 feet with a mean percentage of cut area of 77% measured by ultrasound. Partial Achilles tenotomy may be high risk for the late relapse of deformity in Group I. The highest ICFSG score was obtained in Group III (age more than 3 months), and the score was mainly dependent on the results of radiographic evaluation when weight-bearing. We presume that age at initial treatment of more than 3 months may influence alignment of the tarsal bones after Ponseti method treatment. This may be attributed to reduced response of soft tissue to the Ponseti maneuver due to the decreased viscoelastic properties of the connective tissues along with the infant’s age. In some cases treated using the Ponseti method, we found the mild deformity of residual varus, which was characterized radiographically by a decreased talocalcaneal angle including views in both standing anteroposterior and lateral positions. This is consistent with the results reported by Cooper and Dietz (1995). However, the final Dimeglio score was similar among these 3 groups. We presume that the foot morphological evaluation was not completely consistent with its radiographic assessment when weight-bearing.

Many studies suggested that non-compliance with the FAO brace was closely associated with relapse of the deformity (Ponseti 1996, Dobbs et al. 2004, Morcuende et al. 2004, Dobbs and Gurnett 2009, Zhao et al. 2014b, Liu et al. 2016). Our findings indicated that there is no influence of age at initiation of treatment on brace adherence among 3 groups. Ramirez et al. (2011) reported that there was no significant difference in brace adherence or rate of relapse in patients who began treatment before or after 1 month of age. Zionts et al. (2012) also found no difference in brace adherence between infants who began treatment before and after 3 months of age. In contrast, Bor et al. (2009) found that children who began treatment before 28 days of age had poorer brace compliance than those who began treatment later. In our cases with relapse, the most common type of relapse was identified with adductus and equinus in the present study (Figure 4). This is in contrast to Ponseti (1996), who noted slight equinus and varus deformity as the first appearance in the relapse cases, often without increased adductus and cavus of the forefoot. As Ponseti reported, the relapse was caused by the same pathology that initiated the deformity, and the soft tissue abnormality may be an important cause of the deformity with an excess of collagen synthesis in the Achilles tendon, the posterior tibial tendon, and medial and posterior tarsal ligaments (Ponseti 1996). The retraction of connective tissue of the foot and ankle first induced adductus of the forefoot, followed by equinus deformity due to the kinematic coupling relationship between the tarsal bones. Supposing that the relapse was identified at a very early stage, the recurrence of varus deformity was not often observed in our children. We suppose the retraction of soft issue in the relapse cases to be the initial factor for abnormal alignment of the tarsal bones.

We acknowledge some limitations of our study. The study was retrospective and a relatively small number of cases were included. More randomized controlled trials or large-scale case-control studies are required for further validation.

In summary, treatment initiated between 28 days and 3 months of age produced lower rate of relapse and better clinical outcomes.

YBL and SJL contributed equally to this article. YBL contributed substantially to the acquisition of data and drafted the article. SJL and BY performed the statistical analysis. LZ contributed to the conception and design. DHZ contributed to the case collection in clinical practice.

The authors are grateful to Prof. Weiyu Han from the Clinical Medical Research Center and department of Orthopaedics, Zhujiang Hospital of Southern Medical University, for help in statistical analysis.

*Acta* thanks Ole Rahbek for help with peer review of this study.
